# Impact of Layer Thickness on Mechanical Properties and Surface Roughness of FDM-Printed Carbon Fiber-PEEK Composite

**DOI:** 10.3390/ma19091692

**Published:** 2026-04-22

**Authors:** Getu Koro Megersa, Wojciech Sitek, Agnieszka J. Nowak, Łukasz Krzemiński, Wojciech Kajzer, Daria Niewolik

**Affiliations:** 1 Scientific and Didactic Laboratory of Nanotechnology and Materials Technologies, Silesian University of Technology, 44-100 Gliwice, Poland; agnieszka.j.nowak@polsl.pl (A.J.N.); lukasz.krzeminski@polsl.pl (Ł.K.); 2 Department of Biomechatronics, Faculty of Biomedical Engineering, Silesian University of Technology, 41-800 Zabrze, Poland; wojciech.kajzer@polsl.pl; 3 Department of Physical Chemistry and Technology of Polymers, Faculty of Chemistry, Silesian University of Technology, 44-100 Gliwice, Poland; daria.niewolik@polsl.pl

**Keywords:** CFR-PEEK composites, fused deposition modeling, layer thickness, mechanical properties, surface roughness

## Abstract

Fused deposition modeling (FDM)-based three-dimensional (3D) fabrication offers a viable approach to manufacturing highly customized carbon fiber-reinforced polyether ether ketone (CFR-PEEK) components with complex geometries. However, the mechanical properties and surface roughness of FDM-fabricated parts are strongly influenced by processing parameters, particularly layer thickness. This study investigates the influence of layer thickness (0.1 mm and 0.2 mm) on the surface roughness, crystallinity, mechanical properties, and morphological characteristics of FDM-printed 10% CFR-PEEK specimens. The specimens were characterized using mechanical testing, differential scanning calorimetry (DSC), confocal laser microscopy, X-ray micro-computed tomography (µCT), and scanning electron microscopy (SEM). The results show that specimens printed with a 0.2 mm layer thickness exhibit higher crystallinity and ball indentation hardness while also showing increased surface roughness and porosity, with µCT analysis revealing larger and more spatially clustered voids near the sub-perimeter regions. In contrast, specimens printed with a 0.1 mm layer thickness demonstrate higher tensile strength, elastic modulus, elongation at break, and compressive stress. SEM fractography further indicates improved interlayer bonding and a relatively cohesive fracture surface in specimens printed with a 0.1 mm layer thickness. These findings demonstrate clear layer-thickness-dependent processing–structure–property relationships in FDM-printed CFR-PEEK composites and provide guidance for optimizing printing parameters to achieve improved mechanical performance.

## 1. Introduction

The development of carbon fiber-reinforced polyether ether ketone (CFR-PEEK) composites has expanded the use of high-performance polymers in biomedical, aerospace, and automotive fields [1,2,3]. With growing interest in CFR-PEEK, its fabrication via conventional processing routes poses challenges for producing complex and customer-specific components [2,4]. Over the past decade, additive manufacturing (AM) processes, including selective laser sintering (SLS) and fused deposition modeling (FDM) have emerged as promising alternatives to conventional CFR-PEEK processing methods, including machining, injection molding, and extrusion [4,5]. AM is a layer-wise fabrication process driven by digital instructions and capable of producing complex, lightweight, and customized parts [6,7]. Among AM technologies, FDM has gained increasing attention for manufacturing CFR-PEEK components due to its lower operational costs, simpler setup, reduced material waste through filament-based feeding, improved energy efficiency, and the absence of the complex post-processing steps required in SLS [8,9].

Nevertheless, achieving optimal performance in FDM-printed components remains challenging due to surface irregularities, weak interlayer bonding, and voids [10,11]. These defects are strongly influenced by printing parameters [12,13], printer routines [14], the intrinsic characteristics of the PEEK matrix [15], and the carbon fiber content [3,16]. Consequently, multiple studies have been devoted to analyzing the influence of printing parameters on the mechanical properties of FDM-printed CFR-PEEK composites. For example, Li et al. [17] successfully fabricated 5% CFR-PEEK using FDM and reported that specimens printed in the on-edge (vertical) orientation exhibited higher flexural strength and lower porosity than horizontally printed parts. Gupta et al. [18] and Pagliarulo et al. [19] analyzed the effect of raster angle on the tensile behavior of 10% CFR-PEEK and found that specimens printed at 0° (parallel to the loading direction) achieved the highest tensile strength.

In addition, Yang et al. [20] examined the effect of chamber temperature on the crystallinity and flexural strength of 10% CFR-PEEK, demonstrating that isothermal crystallization and enhanced flexural strength occurred when the chamber temperature exceeded the glass-transition temperature. Higher chamber temperatures have also been associated with reduced porosity and enhanced interlayer bonding [10]. Furthermore, Naganaboyina et al. [21] examined the effects of chamber, nozzle, and platform temperatures on the tensile strength of FDM-fabricated composites and concluded that elevated thermal conditions significantly enhanced interlayer bonding, thereby improving tensile strength.

Among the various FDM printing parameters, layer thickness is one of the critical parameters that significantly influences the surface roughness and mechanical properties of printed parts [11,22]. Owing to the layer-wise deposition principle of additive manufacturing, variations in layer thickness govern interfacial bonding strength and inter-bead void size by altering the effective contact area between adjacent filaments and successive layers [23,24,25,26,27]. In a study by Mutyala et al. [28], a full-factorial experimental design was employed to evaluate the effects of layer thickness, build orientation, and printing speed on the tensile and compressive properties of FFF-printed 30% CFR-PEEK composites. Their results showed that tensile strength was significantly affected by all three parameters and their interactions, whereas compressive strength was primarily governed by the main effects of build orientation and layer thickness, with printing speed showing no statistically significant influence. De Carvalho et al. [29] applied a central composite design to evaluate the influences of layer thickness, printing temperature, and printing speed on the tensile, bending, and impact strengths of 20% CFR-PEEK, demonstrating that tensile strength was most sensitive to layer thickness, while bending and impact strengths were predominantly influenced by printing temperature. A parameter combination involving a 0.1 mm layer thickness, a printing temperature of 385 °C, and a printing speed of 17.5 mm s^−1^ was reported to result in improved overall mechanical properties. In another study, Wang et al. [30] showed that variations in layer thickness have a pronounced effect on the tensile, flexural, and impact properties of FDM-printed CF/PEEK and GF/PEEK composites. Moreover, layer thickness plays a crucial role in controlling inter-filament gaps between adjacent deposited filaments, which can serve as potential sites for crack initiation under mechanical loading, and in determining the surface roughness of printed parts along the direction perpendicular to the build direction [11,31].

Although previous studies have investigated the influence of various FDM printing parameters on the mechanical behavior of CFR-PEEK composites, including tensile properties, flexural strength, and compressive strength [17,18,20,21,28,29,30], the effect of layer thickness on the hardness of CFR-PEEK components fabricated via FDM has not yet been investigated in the literature. In addition, even though the effect of layer thickness on the compressive behavior of CFR-PEEK was examined using design-of-experiments (DOE) approaches in a limited number of studies [28,32], investigating the influence of layer thickness while keeping other parameters constant is critical to understanding how layer thickness governs key microstructural features, such as porosity, which critically influence compressive strength [33]. Furthermore, studies in the literature investigating the influence of layer thickness in FDM-printed CFR-PEEK composites have predominantly focused on mechanical testing [28,29,30,32]. However, a combined investigation of crystallinity, porosity, and mechanical properties is essential to obtain a clearer understanding of how changes in layer thickness affect microstructural features and, consequently, mechanical properties [34]. Moreover, layer thickness influences surface roughness in FDM-fabricated components due to the stair-stepping effect [25]. For CFR-PEEK composites, existing studies have mainly examined surface quality in the context of hybrid additive–subtractive processes, such as dry milling, rather than evaluating the direct influence of layer thickness on as-printed surface roughness [35].

Therefore, to address these gaps, this study investigates the surface roughness, ball indentation hardness, compressive response, and tensile properties of FDM-printed CFR-PEEK composites fabricated with layer thicknesses of 0.1 mm and 0.2 mm. Three-dimensional micro-computed tomography (3D µCT), differential scanning calorimetry (DSC), and scanning electron microscopy (SEM) are employed to characterize porosity, crystallinity, and fracture behavior, respectively, thereby providing further insight into the failure mechanisms of the composites. By integrating these techniques, this study elucidates the effect of layer thickness on microstructural features and the resulting mechanical behavior of CFR-PEEK components.

## 2. Materials and Methods

### 2.1. Printing Materials

The KetaSpire^®^ PEEK CF10 LS1 AM filament, containing 10 wt% carbon fiber reinforcement within the PEEK matrix, was used for specimen preparation. This filament, supplied by 3DGence (Przyszowice, Poland), has a diameter of 1.75 mm, a density of 1.33 g/cm^3^, and a melting temperature of 343 °C [36].

### 2.2. Fabrication of Specimens via 3D Printing

**Figure 1:**
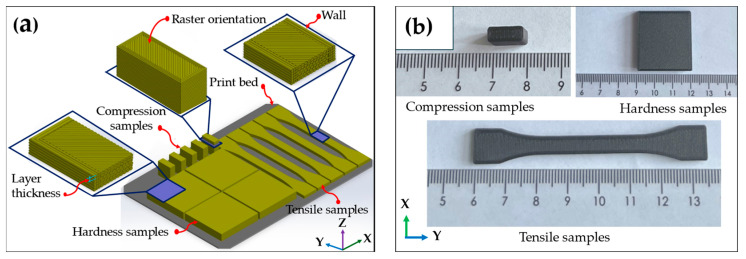
FDM 3D-printed CFR-PEEK samples: (**a**) Forming direction; (**b**) Samples for hardness, compression, and tensile testing.

Three-dimensional CAD models of tensile, compression, and ball indentation hardness specimens were designed using SolidWorks 2024 in accordance with the relevant standards: EN ISO 527-2:2012 for tensile testing (dog-bone-shaped specimens, 75 mm × 5 mm × 2 mm) [37]; PN-EN ISO 604:2003 for compression testing (10 mm × 10 mm × 4 mm blocks) [38,39]; and ISO 2039-1:2001 for ball indentation hardness testing (30 mm × 40 mm × 5 mm plates) [40]. Prior to printing, the CFR-PEEK filament was dried at 150 °C for 4 h to eliminate residual moisture and ensure stable extrusion. The CAD models were exported as STL files, sliced using 3DGence SLICER 4.0 after defining all fabrication parameters, and converted into machine-specific G-code. Specimens were fabricated using an FDM 3D printer (3DGence INDUSTRY F420, 3DGence, Przyszowice, Poland), which is equipped with a maximum build plate temperature of 190 °C, a maximum chamber temperature of 110 °C, and a maximum nozzle temperature of 475 °C. In this study, two groups of specimens were printed using layer thicknesses of 0.1 mm and 0.2 mm, respectively. The printing parameters employed are summarized in Table 1. As illustrated in Figure 1a, specimens for tensile testing were printed in the horizontal build orientation, whereas compression specimens were printed in the vertical build orientation. The 3DGence ESM-10 support material was used to anchor the first layer to the build plate, allowing for easy removal, minimal residue, and reduced warping. After printing, the parts were detached from the platform and air-cooled to room temperature (25 °C) (Figure 1b).

**Figure 2:**
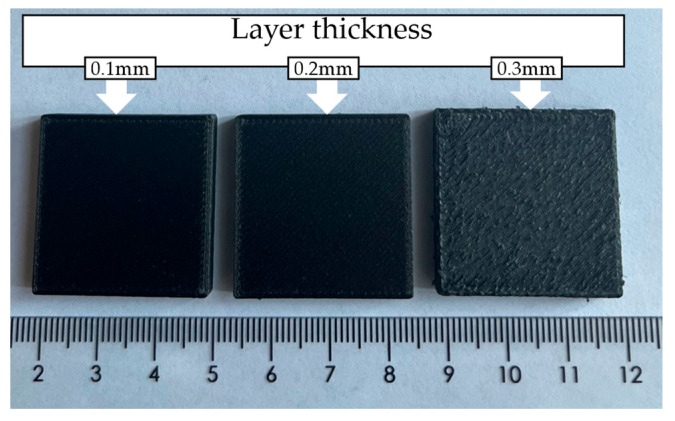
Surface quality comparison of FDM-printed CFR-PEEK specimens fabricated with layer thicknesses of 0.1 mm, 0.2 mm, and 0.3 mm, highlighting pronounced surface irregularities and reduced dimensional accuracy at 0.3 mm.

The layer thicknesses selected for this investigation were determined based on visual observations during initial trial prints using a 0.4 mm nozzle. Three values (0.1 mm, 0.2 mm, and 0.3 mm) were initially printed. As shown in Figure 2, specimens fabricated with a layer thickness of 0.3 mm exhibited pronounced surface irregularities, including non-uniform filament deposition, poor edge definition, and visible inter-bead discontinuities, indicating unstable material deposition and reduced dimensional accuracy [41]. These defects reduce the reliability of both mechanical and surface characterization. Irregular deposition is expected to introduce variability in tensile and compressive responses, while non-uniform surface topology does not meet the requirements of standardized indentation hardness testing (ISO 2039-1) and prevents accurate surface roughness measurement [40]. In addition, the 0.3 mm specimens showed poor repeatability across multiple builds, further limiting their suitability for reproducible experimental evaluation. Consequently, the 0.3 mm condition was excluded from further analysis. In contrast, specimens fabricated at 0.1 mm and 0.2 mm exhibited consistent filament deposition, improved surface quality, and reliable repeatability. This selection is further supported by previous studies [24,42], as well as findings reported in Wang et al. [43], which indicate that when the layer thickness approaches or exceeds approximately half of the nozzle diameter, surface quality deteriorates in high-performance thermoplastics. Similarly, owing to the high viscosity of heat-resistant polymers and their composites, printing speeds above 30 mm/s are not recommended because they adversely affect material flow and print quality [30,43]. Furthermore, all common printing parameters summarized in Table 1 were selected based on values reported in the literature and maintained within the filament supplier’s recommended ranges [29,36,44,45].

### 2.3. DSC Crystallinity Analysis

The thermal behavior of FDM-fabricated CFR-PEEK specimens was evaluated using a differential scanning calorimeter (DSC 3, Mettler-Toledo, Greifensee, Switzerland). The analysis included glass transition temperature, crystallization temperature, melting temperature, and degree of crystallinity. Samples with a mass of approximately 4–5 mg were heated from 25 °C to 400 °C at a heating rate of 10 °C/min, followed by cooling to 25 °C at 5 °C/min. The crystallinity (*X_c_*) of PEEK in the composite was calculated from the first heating cycle according to Equation (1) [46]:

(1) $$X_{c}=\left(\frac{∆H_{f}-∆H_{c}}{(1-wt.\%){∆H_{f}}^{PEEK}}\right)$$

where $∆H_{f}$ represents the melting enthalpy of the composite, $∆H_{c}$ corresponds to the enthalpy of cold crystallization, and ${∆H_{f}}^{\mathrm{PEEK}}$ is the theoretical melting enthalpy of fully crystalline PEEK (130 J/g) [47]. The term wt.% denotes the weight fraction of the reinforcement phase.

### 2.4. Mechanical Test

Mechanical tests were carried out to assess the tensile, compressive, and hardness behavior of the CFR-PEEK specimens. Tensile experiments were performed according to ISO 527-2:2012 (Type 1BA) using dumbbell-shaped specimens [37]. Testing was conducted on a Shimadzu Autograph AGSX universal testing system (Shimadzu Corporation, Kyoto, Japan) equipped with a 10 kN load cell and a mechanical extensometer. A constant crosshead speed of 5 mm/min was applied under ambient conditions. For each layer thickness (0.1 mm and 0.2 mm), five specimens were tested to ensure repeatability. The specimen width and thickness were measured at multiple locations using a Magnusson digital caliper (150 mm) (Limit, Wroclaw, Poland), and the averaged values were used for stress–strain calculations. The data were acquired using TRAPEZIUM X software (version 1.4.5) and further processed in Excel to determine the elastic modulus, elongation at break, and ultimate tensile strength (UTS). Compression testing was performed using a ZWICK Z020 testing machine (ZwickRoell, Ulm, Germany) equipped with a 20 kN load cell. The tests were conducted at a crosshead speed of 1 mm/min following PN-EN ISO 604:2003 standards, with five specimens tested for each layer thickness [38,48]. The compression tests were continued up to 50% strain, as no distinct failure occurred due to progressive deformation and an increase in the effective cross-sectional area [49]. Ball indentation hardness was measured using a Zwick 3106 hardness tester (Zwick GmbH & Co., Ulm, Germany) with a 5 mm diameter steel indenter under a load of 358 N applied for 30 s, in accordance with ISO 2039-1:2001 [40]. For each layer thickness, four specimens were evaluated, with five indentations performed per specimen. The spacing between adjacent indentations was maintained at a minimum of three times the indentation diameter to avoid interaction effects. Hardness values (H, N/mm^2^) were calculated based on the indentation depth.

### 2.5. Morphological Characterization

The top surfaces of the hardness specimens were characterized using a laser confocal microscope (VK-X250 Series, KEYENCE Corp., Osaka, Japan). Measurements were performed using a 5× objective lens. A scanning area of 2.75 × 2.06 mm^2^ was analyzed at five different positions on each specimen, and the average surface roughness parameters were calculated. The three-dimensional surface topography of the 3D-printed CFR-PEEK specimens was also evaluated using the same laser confocal microscope.

The void morphology and void volume distribution of the compression specimens were characterized using X-ray micro-computed tomography (µCT; Nikon XT H 225 ST, Nikon, Minato, Japan). Scans were conducted at an accelerating voltage of 60 kV and a current of 150 µA, with a voxel size of 4 µm. Image reconstruction was carried out using Nikon CT Pro 3D software (version XT 6.13), incorporating beam hardening correction to minimize grayscale inhomogeneity across the specimen cross-section. Reconstructed volumetric datasets were processed in VGStudio Max 2023 (Volume Graphics), where a non-local means noise reduction filter was applied to suppress imaging artifacts while preserving void edge features. Void segmentation was performed using a global grayscale threshold selected based on histogram analysis of a representative volume exhibiting a clear bimodal distribution between the solid material and void phases. The selected threshold was validated by visual comparison of segmented void boundaries against raw grayscale images for a subset of slices. To exclude partial volume artifacts at the resolution limit, only voids larger than three times the voxel size were included in the analysis. The analysis was performed over the entire specimen volume using a consistent region of interest, and all image processing and segmentation parameters were applied uniformly across specimens to ensure comparability. The selected threshold was further verified to provide consistent segmentation of void regions across all specimens.

The fracture morphology of the tensile specimens was analyzed using a field-emission scanning electron microscope (FE-SEM, Gemini Leo 1525, Zeiss, Thornwood, NY, USA) operated at 15 kV. Prior to imaging, specimens were sectioned to the required dimensions and coated with a thin platinum layer to enhance surface conductivity and imaging quality.

### 2.6. Statistical Analysis

Statistical analyses were conducted using OriginPro 2024 (OriginLab Corporation, Northampton, MA, USA). Initially, the normality of all datasets, including mechanical properties and surface roughness parameters, was evaluated using the Shapiro–Wilk test, where *p* > 0.05 indicates no significant deviation from a normal distribution. After verifying the normality assumption, the data were analyzed using a two-sample *t*-test to determine differences between specimens fabricated with layer thicknesses of 0.1 mm and 0.2 mm. A significance level of *p* < 0.05 was adopted for all statistical tests.

## 3. Results

### 3.1. Surface Topography and Roughness

**Figure 3:**
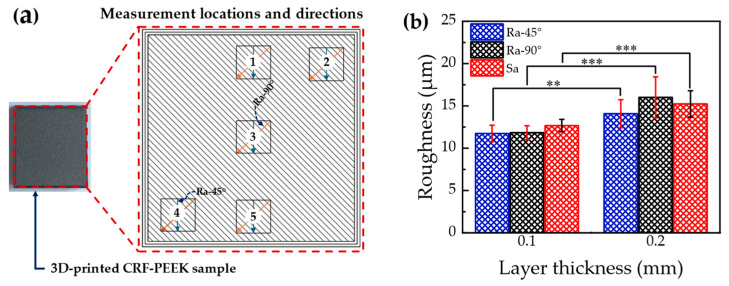
(**a**) Surface roughness measurement locations on the top surface of the 3D-printed CFR-PEEK samples. (**b**) Mean areal and line roughness at different layer thicknesses, with error bars representing the standard deviation. Black rectangles indicate the five areas used for surface roughness measurements. Orange and blue arrows indicate the directions used for line roughness measurements perpendicular to the raster direction (Ra-90°) and at 45° to the raster direction (Ra-45°), respectively. ** and *** indicate statistically significant differences at *p* < 0.01 and *p* < 0.001, respectively (two-sample *t*-test).

The areal and line surface roughness results obtained from five measurement locations (Figure 3a) on the top surfaces of two CFR-PEEK composite specimens per layer thickness (0.1 mm and 0.2 mm), together with the Shapiro–Wilk normality test results for these parameters, are presented in Table A1 (Appendix A). At each of the five measurement locations, the areal surface roughness (Sa) was determined from a scanned area of 2.75 × 2.06 mm^2^. The corresponding line roughness values were then obtained from surface topography data extracted over the same area, along directions perpendicular (Ra-90°) and at 45° (Ra-45°) to the raster orientation. The Shapiro–Wilk test results indicate no significant deviation from normality (*p* > 0.05). Accordingly, two-sample *t*-tests were performed, and the results are graphically presented in Figure 3b and summarized in Table 2. As shown in Figure 3b, the mean Sa for specimens fabricated with a 0.1 mm layer thickness (12.68 µm) is significantly lower (*p* < 0.001) than that for specimens fabricated with a 0.2 mm layer thickness (15.23 µm), representing a reduction of 16.7%. Similarly, the mean Ra-90° (*p* < 0.001) and Ra-45° (*p* = 0.001) values for specimens fabricated with a 0.1 mm layer thickness are significantly lower than those for specimens fabricated with a 0.2 mm layer thickness. Specifically, specimens fabricated with a 0.1 mm layer thickness exhibit mean Ra-90° and Ra-45° values of 11.84 µm and 11.74 µm, respectively, 26.0% lower than the Ra-90° value (16.00 µm) and 16.6% lower than the Ra-45° value (14.07 µm) for specimens fabricated with a 0.2 mm layer thickness.

**Figure 4:**
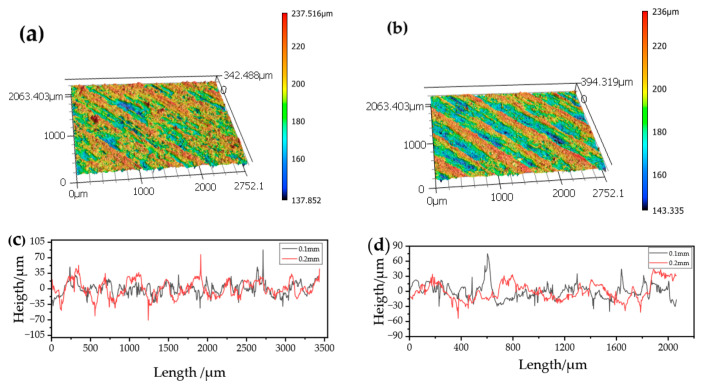
Surface topographies and line profiles of 3D-printed specimens: (**a**) surface topography of the specimen printed with 0.1 mm layer thickness, (**b**) surface topography of the specimen printed with 0.2 mm layer thickness, (**c**) line roughness profile perpendicular to the raster direction (Ra-90°), and (**d**) line roughness profile at 45° to the raster direction (Ra-45°).

Figure 4 illustrates the surface topography and corresponding line roughness profiles of printed specimens acquired at measurement point 3 (Figure 3a) for layer thicknesses of 0.1 mm and 0.2 mm. Both specimens exhibit a distinct surface texture aligned with the raster orientation. The specimen printed with a 0.1 mm layer thickness exhibits comparatively finer and more uniformly distributed height variations in the topography, along with smoother line profiles and reduced peak-to-valley amplitudes. In contrast, the specimen printed with a 0.2 mm layer thickness displays more pronounced surface features, characterized by thicker and more continuous ridges and valleys in the topography and larger height fluctuations in the corresponding line roughness profiles.

### 3.2. Crystallinity of FDM-Printed CFR-PEEK

**Figure 5:**
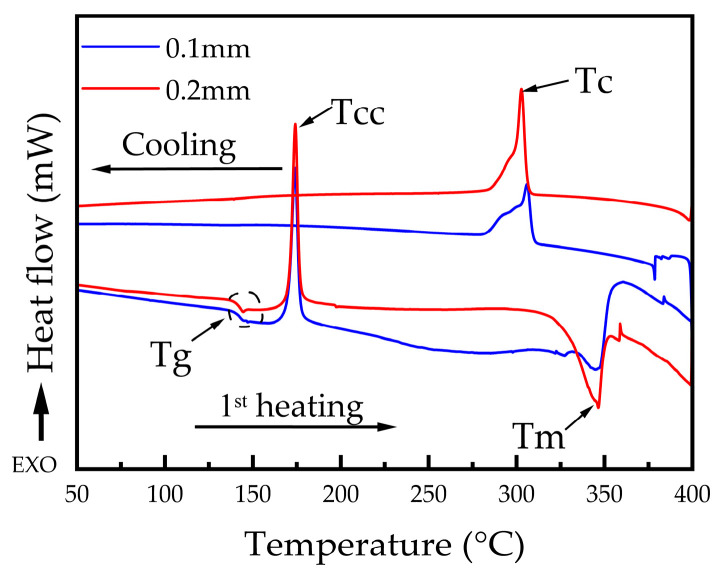
DSC curves of CFR-PEEK specimens fabricated at different layer thicknesses.

The DSC curves of CFR-PEEK printed parts produced with varying layer thicknesses are presented in Figure 5. The main thermal parameters obtained from the DSC analysis are summarized in Table 3, including the glass transition temperature (*T_g_*), crystallization temperature (*T_c_*), cold crystallization temperature (*T_cc_*), melting temperature (*T_m_*), melting enthalpy (Δ*H_m_*), and degree of crystallinity (*X_c_*). As shown in Figure 5, DSC thermograms corresponding to specimens fabricated at different layer thicknesses during heating indicate that both specimens exhibit an exothermic peak around 174 °C and an endothermic peak around 346 °C. A slight decrease in *T_g_* is observed with increasing layer thickness, decreasing from 142.56 °C for specimens fabricated at a layer thickness of 0.1 mm to 141.10 °C for those fabricated at 0.2 mm. No significant shift in the cold-crystallization temperature or melting temperature is observed as the layer thickness varies. Moreover, a higher degree of crystallinity is observed for specimens manufactured with a 0.2 mm layer thickness (9.54%) compared to those manufactured with a 0.1 mm layer thickness (7.42%), as summarized in Table 3.

### 3.3. Mechanical Properties

**Figure 6:**
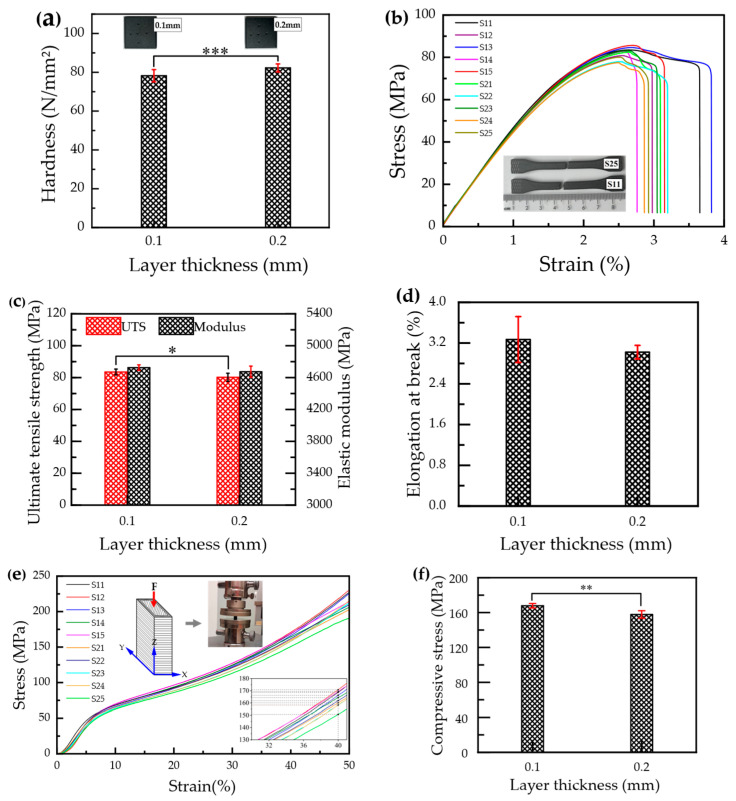
Mechanical properties of FDM 3D-printed CFR-PEEK specimens with different layer thicknesses: (**a**) mean ball indentation hardness, (**b**) tensile stress–strain curves, (**c**) mean UTS and mean elastic modulus, (**d**) mean elongation at break, (**e**) compressive stress–strain curves (the inset shows a magnified view of the curves, with dashed lines indicating the stress values at 40% strain), and (**f**) mean compressive stress, with error bars representing the standard deviation. S11–S15 denote specimens printed with a 0.1 mm layer thickness, whereas S21–S25 denote specimens printed with a 0.2 mm layer thickness. *, **, and *** indicate statistically significant differences at *p* < 0.05, *p* < 0.01, and *p* < 0.001, respectively (two-sample *t*-test).

The results of ball indentation hardness, ultimate tensile strength (UTS), elastic modulus, elongation at break, and compressive stress, together with the Shapiro–Wilk normality test results for all mechanical properties, are presented in Table A2 (Appendix A). The Shapiro–Wilk test results indicate no significant deviation from normality (*p* > 0.05). Accordingly, two-sample *t*-tests were performed for each mechanical property. The results are graphically presented in Figure 6a,c,d,f, and summarized in Table 4. As shown in Figure 6a, the mean ball indentation hardness of specimens printed at a 0.2 mm layer thickness is significantly higher (*p* < 0.001) than that of specimens printed at a 0.1 mm layer thickness. Specifically, specimens printed with a 0.2 mm layer thickness exhibit a mean ball indentation hardness of 82.27 N/mm^2^, which is 5.1% higher than that of specimens printed with a 0.1 mm layer thickness (78.25 N/mm^2^).

The stress–strain response of CFR-PEEK specimens printed at layer thicknesses of 0.1 mm (S11–S15) and 0.2 mm (S21–S25) is illustrated in Figure 6b. While both sets of specimens exhibit a comparable linear elastic regime, their behavior diverges beyond the yield point. Specimens printed at 0.1 mm show a more gradual elastic–plastic transition and sustain plastic deformation to higher strain levels prior to fracture. Conversely, specimens printed at 0.2 mm exhibit more abrupt failure, characterized by a sharper stress drop after reaching the maximum stress and a narrower plastic deformation region. From the tensile stress–strain curves, the UTS and elastic modulus (Figure 6c), as well as elongation at break (Figure 6d), were determined. Specifically, specimens printed with a 0.1 mm layer thickness exhibit mean UTS, elastic modulus, and elongation at break values of 83.48 MPa, 4.725 GPa, and 3.27%, respectively, 4.1% higher than the UTS (80.18 MPa), 1.1% higher than the elastic modulus (4.674 GPa), and 8.3% higher than the elongation at break (3.02%) of specimens fabricated with a 0.2 mm layer thickness. However, the mean differences are statistically significant only for UTS (*p* = 0.047), whereas they are not significant for the elastic modulus (*p* = 0.182) and elongation at break (*p* = 0.271), as summarized in Table 4.

The compressive stress–strain curves of the printed CFR-PEEK specimens are presented in Figure 6e. The compressive response of all specimens, irrespective of layer thickness, is characterized by three distinct deformation stages: an initial nonlinear region at low strains, followed by a quasi-linear region where stress increases steadily with strain, and finally a progressive strain-hardening regime at higher strains without an abrupt stress drop. To compare the effect of layer thickness, the compressive stress at 40% strain was analyzed [49]. As shown in Figure 6f, the mean compressive stress recorded for specimens printed with a 0.1 mm layer thickness (167.86 MPa) is significantly higher (*p* = 0.002) than that of specimens printed with a 0.2 mm layer thickness (157.84 MPa).

### 3.4. Microstructure and Porosity

#### 3.4.1. Porosity

**Figure 7:**
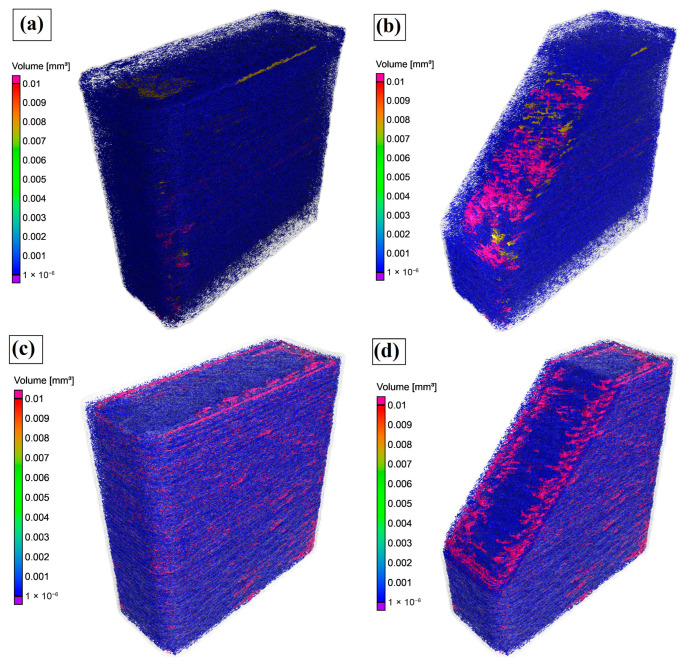
Void distribution in 3D-printed CFR-PEEK specimens: (**a**) 3D CT scan of the specimen fabricated with a 0.1 mm layer thickness, (**b**) sectioned view of the specimen fabricated with a 0.1 mm layer thickness, (**c**) 3D CT scan of the specimen fabricated with a 0.2 mm layer thickness, and (**d**) sectioned view of the specimen fabricated with a 0.2 mm layer thickness.

The void distributions of FDM-printed CFR-PEEK compression specimens fabricated at different layer thicknesses were examined using X-ray µCT, as shown in Figure 7.

The calculated porosity values are summarized in Table 5. It is evident that specimens printed with a 0.1 mm layer thickness have a high population of small voids distributed throughout the specimen volume, together with relatively few large void features (Figure 7a,b). In contrast, specimens printed with a 0.2 mm layer thickness exhibit larger, spatially clustered voids that are predominantly located near the outer wall (sub-perimeter) regions (Figure 7c,d). At the same time, quantitative porosity analysis indicates that an increase in layer thickness leads to higher porosity (Table 5). Notably, the 0.1 mm specimens have a porosity of 6.84%, whereas the 0.2 mm specimens have a porosity of 9.01%, corresponding to a ~32% relative increase.

#### 3.4.2. Fractography

**Figure 8:**
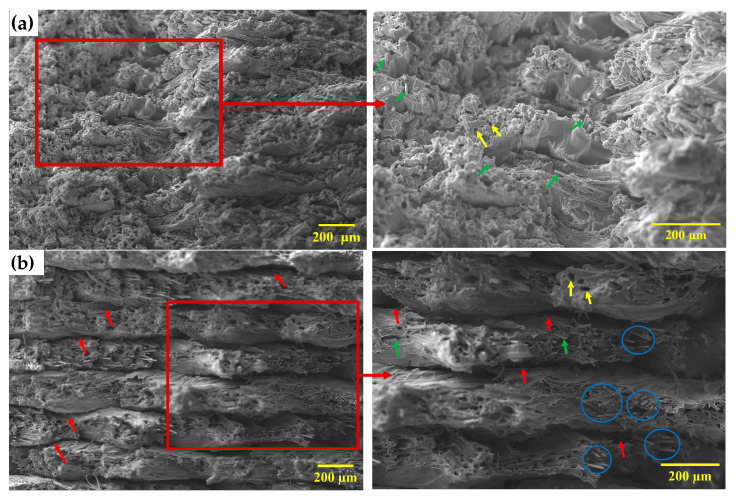
SEM micrographs of fractured tensile specimens at 150× magnification, with corresponding enlarged views of the selected regions at 250× magnification: (**a**) specimen printed with a 0.1 mm layer thickness, and (**b**) specimen printed with a 0.2 mm layer thickness. Red arrows indicate interlayer gaps, yellow arrows indicate elongated fiber pull-out pores, green arrows indicate exposed CF, and blue circles indicate fiber aggregation.

The microstructures of the tensile fracture surfaces of specimens printed with different layer thicknesses are presented in Figure 8. It can be noted that the fracture surface of the specimen printed with a 0.1 mm layer thickness appears relatively compact and continuous, with a rough and uneven morphology. The deposited filaments are closely packed, and the fracture surface shows limited separation between adjacent layers. Carbon fibers are largely embedded within the PEEK matrix, and only a small number of isolated voids and fiber pull-out features are observed (Figure 8a). In contrast, specimens printed with a 0.2 mm layer thickness exhibit pronounced interlayer gaps and clearly visible layer boundaries, resulting in a more layered and discontinuous fracture morphology. Moreover, numerous elongated pores associated with fiber pull-out, regions of localized fiber aggregation, and exposed carbon fiber surfaces are observed (Figure 8b).

## 4. Discussion

The mechanical properties of CFR-PEEK fabricated via FDM primarily depend on interlayer bonding strength [17], porosity [30], crystallinity [3], and fiber–matrix interactions [50]. Interlayer bonding reflects the extent of polymer chain diffusion and entanglement across successive layers, which is critical for load transfer through the printed structure [29]. In contrast, porosity introduces voids and discontinuities between filaments, reducing the effective contact area and acting as stress concentrators. Crystallinity, determined by the degree of molecular ordering and lamellar structure, governs the stiffness and strength of the composite, while fiber–matrix interfacial quality influences load-transfer efficiency and failure mechanisms such as fiber pull-out or debonding [3]. Layer thickness is a key FDM processing parameter that dictates the thermal history and deposition behavior during printing, thereby influencing interlayer bonding, porosity, and crystallinity, and ultimately determining the mechanical properties of printed parts [29]. In this study, CFR-PEEK specimens were fabricated via FDM using layer thicknesses of 0.1 mm and 0.2 mm. The effects of layer thickness on surface roughness and mechanical properties were evaluated. In addition, fractographic, crystallinity, and porosity analyses were performed to examine the underlying mechanisms and relate the observed property variations to layer thickness.

Both areal and line roughness test results indicated that specimens printed with a 0.2 mm layer thickness exhibited higher surface roughness than those printed with 0.1 mm. This is attributed to reduced filament flattening during deposition at higher layer thicknesses [43], as filament flattening depends on the amount of material compressed by the nozzle, which is determined by layer thickness [51]. Along the top surface, roughness is governed by peak-to-valley height variation, which is influenced by bead morphology and degree of flattening [43]. The high melt viscosity of high-performance materials amplifies sensitivity to layer thickness, as flow resistance limits spreading and bonding under reduced compression [27]. Upon deposition, thinner layers promote greater spreading under nozzle compression, flattening the filament to fill gaps between adjacent beads and enhancing inter-bead bonding through diffusion, thereby reducing voids and producing a smoother surface [11]. In contrast, thicker layers reduce nozzle-induced compression, limiting lateral spreading and inter-bead bonding, which increases void formation and surface roughness [11]. In the study by Wang et al. [43], the surface roughness of FDM-printed PEEK parts was analyzed, reporting that Ra measured on the top surface increased from 7.8 μm to 10.2 μm as the layer thickness increased from 0.1 mm to 0.2 mm, and further increased to 15.2 μm as the layer thickness reached 0.25 mm. Similarly, Gao et al. [52] reported that Ra measured on the top surface of FDM-printed PEEK parts along the direction perpendicular to the raster angle increased from 11.43 μm to 14.92 μm as the layer thickness increased from 0.1 mm to 0.2 mm, and further increased to 15.59 μm as the layer thickness reached 0.25 mm. Although these studies investigated neat PEEK, their findings are consistent with the present study, in which specimens printed with a 0.2 mm layer thickness exhibited higher surface roughness than those printed at 0.1 mm. However, the magnitude of roughness observed in the present study is comparatively higher, with Ra values of 11.84 μm (0.1 mm) and 16.00 μm (0.2 mm), measured perpendicular to the raster angle. This behavior is attributed to the higher viscosity of CFR-PEEK composites resulting from fiber incorporation, which leads to greater resistance to flow within the nozzle; hence, during deposition, the nozzle interacts with the previously deposited material, producing irregular deposition tracks, which increases surface roughness [53]. In addition, thicker layers are associated with larger interlayer gaps and more pronounced stratification along the build direction [45]. These features promote stress concentration at interfacial regions and may contribute to earlier failure under mechanical loading compared with specimens printed using thinner layers [54].

The degree of crystallinity is a key factor that influences the properties of semi-crystalline polymer composites [26]. In FDM printing, layer thickness is one of the non-thermal parameters that can alter the thermal history when other processing conditions are held constant [34]. In this study, higher layer thickness corresponds to higher crystallinity. In particular, the crystalline content increased from 7.42% for specimens printed at 0.1 mm to 9.54% for those printed at 0.2 mm. This behavior can be attributed to the larger volume of molten material associated with thicker layers, which increases thermal inertia and promotes prolonged heat retention [55]. As a result, the cooling rate is reduced, allowing more time for polymer chains to reorganize into ordered crystalline structures [34,55]. In contrast, thinner layers possess lower thermal mass and a higher surface-area-to-volume ratio, which enhances heat dissipation and increases the cooling rate [56]. This limits polymer chain organization into ordered crystalline structures, resulting in lower crystallinity [57]. However, the crystallinity values remain relatively low for both layer thicknesses, and the variation between them is minimal. In contrast, Lu et al. [34] reported a more pronounced dependence of crystallinity on layer thickness in CFR-PEEK fabricated via screw extrusion, where crystallinity increased from 0.15 mm to 0.25 mm and subsequently decreased at higher layer heights up to 0.40 mm. This difference can be attributed to the distinct processing conditions in screw extrusion, including continuous material feeding, longer melt residence time, and higher extrusion forces, which result in different thermal histories compared to filament-based FDM. Moreover, DSC thermograms exhibited distinct exothermic peaks associated with cold crystallization, which are indicative of incomplete crystallization during deposition due to rapid cooling and kinetic constraints. This behavior is attributed to the steep thermal gradient between the nozzle and the build chamber, which promotes rapid cooling. As a result, a significant fraction of the polymer solidifies in a metastable amorphous state and crystallizes only upon reheating during DSC analysis [26].

The tensile and compressive test results indicated that specimens printed with a layer thickness of 0.1 mm exhibited higher UTS (83.48 MPa) and compressive stress (167.86 MPa) than those printed with 0.2 mm, with statistically significant differences observed for both properties, whereas the changes in elastic modulus and elongation at break were small and insignificant. Although higher strength and stiffness of semicrystalline composites like CFR-PEEK are typically associated with increased crystallinity [53], this trend was not observed in the present study, as higher crystallinity was found in specimens printed with a 0.2 mm layer thickness. This suggests that the differences in UTS and compressive stress can be attributed to variations in interlayer bonding and porosity associated with layer thickness [58]. Interlayer bonding in FDM-printed parts is governed by the degree of filament compression and interfacial contact during deposition [30]. A larger layer thickness increases the nozzle-to-surface distance, which reduces the compression applied to the extruded filament and limits its lateral spreading. This diminishes the effective contact area between adjacent filaments and successive layers, restricting polymer chain diffusion across interfaces [51]. The result is weakened interlayer adhesion, increased porosity, and pronounced layer stratification, all of which compromise interfacial shear strength and load-transfer efficiency, ultimately reducing mechanical strength [30,59]. Conversely, printing with thinner layers amplifies nozzle-induced compression of the deposited filament, increasing the interfacial contact area and promoting improved fusion between adjacent filaments and successive layers [58]. This facilitates stronger inter-filament and interlayer bonding, as well as a denser microstructure with reduced porosity. Consequently, parts fabricated with thinner layers exhibit greater resistance to fracture under mechanical loading [30,59].

Moreover, the present findings are supported by previous studies that investigated different material systems. El Magri et al. [25] investigated FDM-printed PEEK specimens and reported that increasing the layer thickness from 0.10 mm to 0.20 mm led to reductions of 2.8% in tensile strength, 6% in elongation at break, and 8.6% in Young’s modulus. Similarly, Sikder et al. [59] studied the effect of layer thickness on the mechanical properties of FDM-printed PEEK and demonstrated that a lower layer thickness (0.1 mm) resulted in superior tensile, compressive, and flexural strengths, whereas higher layer heights degraded mechanical performance. This trend is also evident in the study by Sonaye et al. [4], who fabricated dental implants using FDM-printed PEEK and reported compressive strength increases of 24.1% when decreasing the layer thickness from 0.3 mm to 0.2 mm and a further increase of 17.6% when reducing it from 0.2 mm to 0.1 mm.

Even though the compressive stress of FDM-printed CFR-PEEK was significantly influenced by layer thickness, the stress–strain response, irrespective of layer thickness, consistently exhibited three distinct regions: an initial nonlinear region, followed by a quasi-linear region, and finally a progressive strain-hardening regime without a distinct yield point. This strain-hardening behavior is attributed to the progressive closure of pores between layers during compression, which leads to material densification and an increase in effective load-bearing capacity [60]. This mechanism is enabled by the vertical build orientation of the compression specimens, which orients the interlayer interfaces perpendicular to the applied compressive load, thereby promoting void closure rather than opening [49,61]. In contrast, under tensile loading, the applied load acts along the interlayer regions, directly stressing the vulnerable interlayer defects where cracks initiate easily [61]. Despite tensile properties being directly dependent on interlayer bonding, compressive stress showed greater sensitivity to changes in layer thickness [59]. Notably, the enhancement in compressive stress (a difference of 10.02 MPa) was greater than that observed for UTS (a difference of 3.30 MPa). This is because compression specimens were printed with a height of 10 mm, whereas tensile specimens had a thickness of 2 mm. Consequently, compression specimens contained a greater number of layers, meaning the effect of layer thickness was multiplied across many interfaces, leading to more prominent changes in mechanical performance [58]. In essence, a higher number of layers (achieved with smaller layer thickness) enhances compressive stress through improved interfacial bonding, greater structural continuity, and more efficient load distribution across numerous interfaces, whereas a lower number of layers (resulting from larger layer thickness) degrades performance due to reduced load-transfer efficiency, increased void content, and localized stress concentrations [39,59].

In contrast to the tensile and compressive properties observed in this study, ball indentation hardness was higher for specimens printed with a 0.2 mm layer thickness (82.27 N/mm^2^) than for those printed with 0.1 mm (78.25 N/mm^2^). This finding is supported by the study of Kuchampudi et al. [62], who demonstrated that layer thickness significantly affects hardness, with the highest hardness observed for specimens printed with a 0.2 mm layer thickness in FDM-printed PETG composites reinforced with aramid fibers. In contrast to the present findings, Borah and Chandrasekaran [63] reported that the Rockwell hardness of FDM-printed PEEK increases with decreasing layer thickness, with the maximum hardness obtained with a 0.1 mm layer thickness. They attributed this behavior to reduced void content and enhanced compactness of the printed structure at smaller layer heights, which increases resistance to indentation. On the other hand, Prechtel et al. [64] showed that the hardness of FDM-printed PAEK materials is not significantly affected by layer thickness, as comparable Martens hardness values were obtained for layer heights between 0.1 and 0.3 mm. Similarly, Pulipaka et al. [45] reported that layer height does not have a statistically significant effect on the indentation hardness of FDM-printed PEEK. The discrepancies between the present findings and previous reports may be attributed to the fact that most earlier studies investigated unfilled semi-crystalline PEEK [45,63], whereas the present study investigates a CFR-PEEK composite reinforced with 10% short carbon fibers. In the present CFR-PEEK composite, specimens printed with a 0.2 mm layer thickness are expected to contain a higher number of carbon fibers per lamina, predominantly aligned along the raster direction due to the increased layer thickness, compared with specimens printed with a 0.1 mm layer thickness. The higher content of stiff carbon fibers aligned along the raster direction within each lamina may enhance resistance to localized plastic deformation under ball indentation. Furthermore, at higher layer thicknesses, each deposited lamina contains a larger continuous material volume, which may promote more uniform local load distribution during indentation and thereby increase resistance to penetration [65,66]. Additionally, ball indentation hardness exhibited a positive correlation with crystallinity, which may contribute to the higher ball indentation hardness observed in specimens printed with a 0.2 mm layer thickness [67].

Voids affect mechanical properties by acting as stress concentrators [24]. During FDM, the layer-by-layer deposition process introduces internal pores and voids, which significantly influence the mechanical performance of printed parts [68]. The size, shape, and spatial distribution of these voids are governed by processing parameters and directly affect the mechanical response [68]. In this study, X-ray µCT analysis showed that specimens printed with a 0.1 mm layer thickness contained a high population of small, relatively uniformly distributed voids, whereas specimens printed with a 0.2 mm layer thickness exhibited larger and more spatially clustered pores, particularly near sub-perimeter regions. The total porosity was higher in specimens printed with a 0.2 mm layer thickness (9.01%) compared to those printed with a 0.1 mm layer thickness (6.84%). This phenomenon is explained by the increase in nozzle-to-surface distance associated with thicker layers during printing, which reduces filament compression and limits its lateral spreading, particularly in the regions between infill and perimeter paths, as well as between adjacent deposited filaments. This leads to incomplete interfacial contact, thereby promoting void formation in sub-perimeter regions and along deposition paths [69].

The tensile fracture surface morphology of 3D-printed specimens further elucidates the underlying causes of the reduced tensile properties associated with higher layer thickness. The specimen printed with a 0.1 mm layer thickness exhibits relatively compact and continuous interlayer regions with limited separation, whereas the 0.2 mm specimen shows more pronounced interlayer gaps and clearly distinguishable layer boundaries. This suggests that reducing layer thickness promotes more effective interlayer fusion, while increasing layer thickness is associated with lack-of-fusion defects that disrupt load-transfer continuity and reduce the effective load-bearing area [30]. Furthermore, carbon fibers are largely embedded within the matrix, with only limited pull-out features observed in specimens printed with a 0.1 mm layer thickness. In contrast, specimens printed with a 0.2 mm layer thickness exhibit more frequent and elongated pull-out pores, along with exposed fibers. This indicates that specimens printed with a 0.2 mm layer thickness are associated with weaker fiber–matrix interfacial bonding, likely due to fiber aggregation along the raster direction in thicker layers. Such aggregation promotes the formation of void-rich regions at the fiber–matrix interfaces and within fiber clusters, which are difficult for the matrix to fully infiltrate during the printing process [3]. Since the specimens were printed with a 45/−45° raster orientation, the applied tensile load is oriented off-axis relative to the raster direction, resulting in a combination of normal and shear stress components [70]. This influences crack propagation along raster paths and may contribute to interfacial debonding and fiber pull-out, particularly in the presence of weak fiber–matrix bonding and voids. In specimens printed with a 0.2 mm layer thickness, cracks tend to propagate along interlayer gaps, fiber–matrix interfaces, and void-rich regions, suggesting that these defects act as preferred paths for fracture. This crack propagation behavior indicates that ineffective stress transfer between the matrix and fibers, together with discontinuities at interlayer regions, reduces the ability of the composite to sustain tensile loading, leading to premature failure in specimens printed with larger layer thickness [71].

In summary, thinner layers (0.1 mm) enhance filament flattening, interlayer bonding, and load transfer while reducing porosity, resulting in superior tensile and compressive strengths compared to thicker layers (0.2 mm). In contrast, thicker layers increase surface roughness, porosity, and fiber aggregation, leading to weaker fiber–matrix bonding and reduced mechanical properties despite higher crystallinity. Compressive strength exhibits greater sensitivity to layer thickness than tensile strength due to the larger number of layers in taller compression specimens, whereas hardness shows an opposing trend, being higher for thicker layers. However, it is important to acknowledge the limitations of this study. The analysis is limited to two practically relevant layer thicknesses (0.1 mm and 0.2 mm), selected based on printability and surface quality considerations. Therefore, the results should be interpreted as a comparative assessment rather than a full parametric optimization. Future work should investigate additional layer thickness levels within the printable and stable processing window to establish more comprehensive process–structure–property relationships in FDM-printed CFR-PEEK composites.

## 5. Conclusions

In this study, the effect of layer thickness on the surface roughness, crystallinity, and mechanical properties of FDM-printed CFR-PEEK composites was investigated by comparing specimens fabricated with layer thicknesses of 0.1 mm and 0.2 mm. It was found that increasing the layer thickness resulted in higher surface roughness, with the areal surface roughness (Sa) increasing from 12.68 µm to 15.23 µm and the line roughness values (Ra-90° and Ra-45°) increasing by 35.2% and 19.9%, respectively, while the degree of crystallinity increased from 7.42% to 9.54%. The mechanical properties exhibited clear layer-thickness-dependent variations. Specimens printed with a 0.1 mm layer thickness exhibited higher ultimate tensile strength, compressive stress at 40% strain, and elongation at break, with the UTS increasing from 80.18 MPa to 83.48 MPa, the compressive stress increasing from 157.84 MPa to 167.86 MPa, and the elongation at break increasing from 3.02% to 3.27%. In contrast, specimens printed with a 0.2 mm layer thickness exhibited higher ball indentation hardness, which increased from 78.25 N/mm^2^ to 82.27 N/mm^2^. X-ray µCT analysis showed that increasing the layer thickness from 0.1 mm to 0.2 mm resulted in higher porosity, accompanied by a transition in void morphology from small, uniformly distributed pores to larger, spatially clustered voids concentrated near the sub-perimeter regions. Fractographic observations indicated that specimens printed with a 0.1 mm layer thickness exhibited a more compact fracture morphology with fewer interlayer gaps, whereas specimens printed with a 0.2 mm layer thickness exhibited more pronounced interlayer separation, exposed fibers, and fiber pull-out features. Overall, a layer-thickness-dependent processing–microstructure–property relationship was identified for FDM-printed CFR-PEEK composites under the investigated processing conditions. Reducing the layer thickness resulted in lower surface roughness, higher tensile properties, and higher compressive stress, whereas increasing the layer thickness resulted in higher crystallinity, higher ball indentation hardness, and higher porosity. To further enhance the mechanical properties of FDM-printed CFR-PEEK fabricated with lower layer thickness, future work should evaluate the effects of post-processing heat treatment at different temperatures and employ machine-learning-based methods for multi-parameter optimization.

## Figures and Tables

**Table 1:** Printing parameters for test samples.

Parameter	Setting
Layer thickness	0.1 mm, 0.2 mm
Nozzle diameter	0.4 mm
Bead width	0.4 mm
Nozzle temperature	405 °C
Platform temperature	190 °C
Chamber temperature	80 °C
Printing speed	24 mm/s
Infill density	100%
Raster angle	+45°/−45°
Perimeter walls	3
Overlap	0
Build orientation	Flat (tensile and hardness); Vertical (compression)

**Table 2:** Results of two-sample *t*-tests comparing areal and line surface roughness parameters at layer thicknesses of 0.1 mm and 0.2 mm.

Parameters	Layer Thickness (mm)	Mean ± SD	*t*-Statistic	*p*-Value
Sa (µm)	0.1	12.68 ± 0.73	−4.70	<0.001
	0.2	15.23 ± 1.56		
Ra-90° (µm)	0.1	11.84 ± 0.82	−5.15	<0.001
	0.2	16.00 ± 2.43		
Ra-45° (µm)	0.1	11.74 ± 0.97	−3.84	0.001
	0.2	14.07 ± 1.65		

*n* = 10 measurements per group (2 specimens × 5 locations); degrees of freedom (*df*) = 18; SD is the standard deviation; Sa is the areal surface roughness; Ra-90° is the line roughness measured perpendicular to the raster orientation; Ra-45° is the line roughness measured at 45° to the raster orientation; *t* is the *t*-statistic; *p* is the significance level of the two-sample *t*-test.

**Table 3:** DSC results from the first heating–cooling cycle of CFR-PEEK specimens printed at different layer thicknesses.

Layer Thickness (mm)	*T_g_* (°C)	*T_cc_* (°C)	*T_m_* (°C)	*T_c_* (°C)	*H_cc_* (J/g)	*H_f_* (J/g)	*X_c_* (%)
0.1	142.56	174.06	346.97	305.98	20.10	28.78	7.42
0.2	141.10	174.28	346.20	302.90	20.91	32.07	9.54

*T_g_* is the glass transition temperature; *T_cc_* is the cold crystallization temperature; *T_m_* is the melting temperature; *T_c_* is the crystallization temperature; *H_cc_* is the enthalpy of cold crystallization; *H_f_* is the enthalpy of fusion; *X_c_* is the degree of crystallinity.

**Table 4:** Results of two-sample *t*-tests comparing mechanical properties of CFR-PEEK specimens fabricated with layer thicknesses of 0.1 mm and 0.2 mm.

Properties	Layer Thickness (mm)	Mean ± SD	*t*-Statistic	*p*-Value
Ball indentation hardness (N/mm^2^)	0.1	78.25 ± 3.13	−4.80	<0.001
	0.2	82.27 ± 2.07		
UTS (MPa)	0.1	83.48 ± 1.84	2.35	0.047
	0.2	80.18 ± 2.54		
Elastic modulus (GPa)	0.1	4.725 ± 0.035	1.46	0.182
	0.2	4.674 ± 0.070		
Elongation at break (%)	0.1	3.27 ± 0.45	1.18	0.271
	0.2	3.02 ± 0.13		
Compressive stress (MPa)	0.1	167.86 ± 2.54	4.53	0.002
	0.2	157.84 ± 4.24		

*n* = 20 measurements per group and degrees of freedom (*df*) = 38 for ball indentation hardness; *n* = 5 measurements per group and *df* = 8 for UTS, elastic modulus, elongation at break, and compressive stress. SD is the standard deviation; *t* is the *t*-statistic; *p* is the significance level of the two-sample *t*-test.

**Table 5:** Porosity content of FDM 3D-printed samples based on X-ray µCT analysis.

Layer Thickness (mm)	Total Volume (mm^3^)	Material Volume (mm^3^)	Porosity (%)
0.1	344.916	321.32	6.84
0.2	357.62	325.42	9.01

## Data Availability

The original contributions presented in this study are included in the article. Further inquiries can be directed to the corresponding authors.

## Appendix A

**Table A1:** Surface roughness results and Shapiro–Wilk normality test results for CFR-PEEK specimens.

**Surface Roughness (Sa, µm)**									
**Layer** **Thickness (mm)**	**Samples**	**Measurement Locations**						**Shapiro–Wilk Normality Test**	
		**P1**	**P2**	**P3**	**P4**	**P5**	**Mean ± SD**	***W***	***p*** **-Value**
0.1	1	11.91	12.63	12.24	11.81	12.54	12.68 ± 0.73	0.92	0.36
	2	13.52	14.18	13.07	12.36	12.53			
0.2	1	15.79	14.73	18.56	14.83	17.15	15.23 ± 1.56	0.87	0.09
	2	13.74	13.48	14.72	14.47	14.85			
**Line Roughness (Ra-90°, µm)**									
**Layer** **Thickness (mm)**	**Samples**	**Measurement Locations**						**Shapiro–Wilk Normality Test**	
		**P1**	**P2**	**P3**	**P4**	**P5**	**Mean ± SD**	***W***	***p*** **-Value**
0.1	1	11.96	11.05	11.62	12.65	12.20	11.84 ± 0.82	0.97	0.92
	2	13.23	10.87	10.67	12.39	11.71			
0.2	1	14.27	16.09	17.66	15.13	21.80	16 ± 2.43	0.85	0.06
	2	13.35	14.50	15.21	14.79	17.25			
**Line Roughness (Ra-45°, µm)**									
**Layer** **Thickness (mm)**	**Samples**	**Measurement Locations**						**Shapiro–Wilk Normality Test**	
		**P1**	**P2**	**P3**	**P4**	**P5**	**Mean ± SD**	***W***	***p*** **-Value**
0.1	1	11.03	11.56	10.20	13.76	11.45	11.74 ± 0.97	0.96	0.74
	2	11.56	12.28	11.12	11.84	12.61			
0.2	1	12.84	15.17	16.19	11.50	16.81	14.07 ± 1.65	0.98	0.97
	2	14.36	13.72	14.28	13.39	12.47			

P1 is measurement location 1, P2 is measurement location 2, P3 is measurement location 3, P4 is measurement location 4, and P5 is measurement location 5; SD is standard deviation; *W* is the Shapiro–Wilk statistic; *p* is the *p*-level of significance for Shapiro–Wilk test.

**Table A2:** Mechanical properties and Shapiro–Wilk normality test results for CFR-PEEK specimens.

**Ball Indentation Hardness (N/mm^2^)**								
**Layer Thickness (mm)**	**Indentation Measurements**						**Shapiro–Wilk Normality Test**	
	**H1**	**H2**	**H3**	**H4**	**H5**	**Mean ± SD**	***W***	***p*** **-Value**
0.1	71.4	84.3	76	80.1	76.6	78.25 ± 3.13	0.941	0.247
0.1	76.9	79.8	79.8	79.8	78.1			
0.1	79.1	78.8	80.1	79.8	78.8			
0.1	79.4	76.6	82.9	73	73.6			
0.2	80.1	80.1	82.2	80.4	84	82.27 ± 2.07	0.941	0.253
0.2	80.8	83.2	82.5	78.5	85.1			
0.2	78.5	84.3	84	83.2	84.7			
0.2	85.1	80.8	83.6	81.8	82.5			
**Ultimate Tensile Strength (MPa)**								
**Layer Thickness (mm)**	**Samples**						**Shapiro–Wilk Normality Test**	
	**1**	**2**	**3**	**4**	**5**	**Mean ± SD**	***W***	***p*** **-Value**
0.1	83.5	80.8	84.6	82.8	85.6	83.48 ± 1.84	0.98	0.936
0.2	82.4	77.9	83.1	77.5	80.1	80.18 ± 2.54	0.897	0.396
**Elastic modulus (GPa)**								
**Layer Thickness (mm)**	**Samples**						**Shapiro–Wilk normality test**	
	**1**	**2**	**3**	**4**	**5**	**Mean ± SD**	***W***	***p*** **-value**
0.1	4.76	4.69	4.69	4.76	4.72	4.72 ± 0.04	0.887	0.34
0.2	4.72	4.61	4.74	4.59	4.70	4.67 ± 0.07	0.884	0.33
**Elongation at Break (%)**								
**Layer Thickness (mm)**	**Samples**						**Shapiro–Wilk Normality Test**	
	**1**	**2**	**3**	**4**	**5**	**Mean ± SD**	***W***	***p*** **-value**
0.1	3.65	2.98	3.82	2.76	3.15	3.27 ± 0.45	0.93	0.60
0.2	3.09	3.19	3.04	2.86	2.92	3.02 ± 0.13	0.97	0.88
**Compressive Stress (MPa)**								
**Layer Thickness (mm)**	**Samples**						**Shapiro–Wilk Normality Test**	
	**1**	**2**	**3**	**4**	**5**	**Mean ± SD**	***W***	***p*** **-Value**
0.1	168.66	170.76	166.28	164.36	169.26	167.86 ± 2.54	0.96	0.81
0.2	160.07	161.85	158.23	158.32	150.73	157.84 ± 4.24	0.85	0.20

H1 is indentation measurement 1, H2 is indentation measurement 2, H3 is indentation measurement 3, H4 is indentation measurement 4, and H5 is indentation measurement 5; SD is standard deviation; *W* is the Shapiro–Wilk statistic; *p* is the *p*-level of significance for the Shapiro–Wilk test.
